# Quality of life in diverticular disease: translation and validation of the Danish version of the diverticulitis quality of life instrument (DV‑QOL)

**DOI:** 10.1007/s00384-025-04911-z

**Published:** 2025-05-14

**Authors:** Helene R. Dalby, Katrine J. Emmertsen

**Affiliations:** 1 Department of Surgery, Randers Regional Hospital, Skovlyvej 17, 8930 Randers, Denmark; 2 Department of Clinical Medicine, Aarhus University, Aarhus, Denmark

**Keywords:** DV-QOL, Diverticulosis, Diverticular disease, Quality of life, PROM, Validation

## Abstract

**Purpose:**

This study aimed to translate and validate the Danish version of the DV-QOL questionnaire, originally developed in 2015, to assess the impact of diverticular disease on quality of life (QOL) in Danish-speaking patients with diverticulosis.

**Methods:**

Following international standards, the DV-QOL was translated. A cross-sectional survey was conducted in 2023 with Danish-speaking subjects. The survey included the Danish DV-QOL, an anchor QOL question, and the EuroQol visual analogue scale. Psychometric properties were evaluated for validity, internal consistency, and the ability to identify significant impacts on QOL.

**Results:**

The validation cohort included 16,766 subjects. The DV-QOL score showed a strong correlation with overall QOL (*p* < 0.001) and high discriminative validity (*p* < 0.001). Reliability was confirmed with an inter-item correlation of 0.41 and a Cronbach’s α of 0.92. The score accurately identified patients with a significant impact of bowel function on QOL, achieving 82% sensitivity and 79% specificity.

**Conclusion:**

The Danish DV-QOL is a valid and reliable tool for measuring diverticular disease-specific QOL, beneficial for both clinical and research applications in understanding the impact of the disease and patient outcomes.

**Supplementary Information:**

The online version contains supplementary material available at 10.1007/s00384-025-04911-z.

## Introduction

Health-related quality of life (QOL) is the multidimensional concept of how disease and treatment affect the physical, psychological, mental, and social aspects of life as subjectively perceived by the individual [1, 2]. The impact of diverticular disease on QOL is increasingly acknowledged [3–8], and current guidelines for diverticular disease management recognise QOL as a fundamental consideration alongside clinical outcomes [9, 10].

Patient-reported outcome measures (PROMs) are standardised instruments designed to collect information from the patient’s perspective on symptoms, functional status, and QOL [11]. PROMs offer a patient-centred approach to measuring health and essential implications in clinical decision-making.

Beyond bowel symptoms, diverticular disease has been described to be associated with mental and psychosocial distress [5, 12]. To facilitate the evaluation and monitoring of relevant functional outcomes in diverticular disease, the diverticulitis QOL instrument (DV-QOL) was developed in 2015 [4]. The instrument assesses four domains: physical symptoms, concerns, feelings, and behavioural changes.

The DV-QOL was developed for patients with symptomatic, uncomplicated diverticular disease following acute diverticulitis, and a similar cohort was used to establish a patient-acceptable symptoms state (PASS) score [4, 25]. The score has been validated in German [31] and Italian [32] cohorts comprising patients with recurrent acute diverticulitis. To date, few studies have examined QOL among individuals with diverticulosis diagnosed incidentally, without a history of acute diverticulitis, and little is known about the symptom burden in these individuals.

Currently, there is no available diverticular disease-specific instrument to investigate QOL in Danish-speaking subjects. This study, therefore, aimed primarily to evaluate the psychometric properties of the Danish version of the DV-QOL instrument following its formal translation from English. In addition, we assessed the sensitivity and specificity of the instrument in identifying patients with a significant impact of diverticular disease on QOL. Secondarily, the study aimed to explore whether the presence of diverticulosis alone, without a history of clinical episodes, may be associated with impaired QOL.

## Materials and methods

This cross-sectional study is reported following The Strengthening in Reporting of Observational Studies in Epidemiology (STROBE) Statement guidelines for reporting cross-sectional studies [13]. Terminology of the properties of the instruments follows the COSMIN taxonomy where applicable [14]. Written informed consent was obtained from all participants. The study was registered at the Danish Data Protection Agency in the Central Denmark Region (record no. 1–16-02–), and the disclosure of information was approved by the Central Denmark Region (record no. 1–45–70–19–22).

### Translation

According to previously published recommendations, a forward–backwards translation from the original English Version of the DV-QOL into Danish was performed [15, 16]. In the first step, two independent native Danish speakers, one of whom was a medical doctor, performed a forward translation from the original English version into Danish. The translators discussed any discrepancies between the two versions until a final consensus was reached. In the second step, the consensus version was back-translated into English by a native English speaker without prior knowledge of the original version. The back-translations were conducted to verify whether the original meaning of each question was accurately preserved. A native English-speaking editor reviewed the pre-final version of the Danish DV-QOL and subsequently consulted with the investigators, who are experts in medicine, to identify any discrepancies; the final Danish version was then established.

A random sample of 100 subjects from the study cohort was invited to participate in the pilot testing of the Danish DV-QOL, of whom 53 (53%) responded. Respondents were asked to provide written or oral feedback regarding the clarity and comprehensibility of the questionnaire items. No substantial difficulties in understanding the questions were reported, and consequently, no modifications were made to the final version.

### Validation cohort

The validation cohort included responders from the DIVIPACT cohort. The DIVIPACT cohort comprises respondents to a cross-sectional online questionnaire survey conducted in the Central Denmark Region (comprising five public hospitals and ~ 1.3 million residents) in April and May 2023. The Danish healthcare system provides free, tax-supported healthcare for all citizens. All hospital contacts in Denmark are registered with one primary diagnosis and potentially multiple secondary diagnoses coded using the International Classification of Diseases, Tenth Revision (ICD-10) [17]. Subjects eligible for the DIVIPACT cohort were identified in August 2022 based on a primary or secondary diagnosis of diverticulosis (ICD-10 codes K57.2–9) during an inpatient or outpatient hospital contact in the Central Denmark Region between 2010 and 2022. Those diagnosed with colorectal cancer or dementia, deceased subjects, and subjects unable to receive digital mail from public Danish authorities were excluded.

### Data collection

All Danish residents aged ≥ 15 years with a civil person registration (CPR) number are required to receive digital mail from public authorities unless exempt, and 88% of the Danish population use the secure digital mailing system e-Boks [18, 19]. Eligible subjects received an invitation letter in e-Boks, including a link to the online questionnaire and links to webpages with more information about the study and data handling and security [20, 21].

#### The questionnaire

The questionnaire included self-reported health factors and questions on counselling with their general practitioner due to diverticular disease flare-ups, as well as the Danish version of the DV-QOL, an anchor QOL question, and the EuroQOL (EQ) visual analogue scale (VAS) score from the EQ 5 Dimension survey [22].

#### Record linkage to the Danish health registries

Using the CPR numbers, individual responses could be linked to personal medical records. After thorough information, participants were asked to consent to data linkage. Data extraction for participants who consented was conducted in February 2024.

### Validation cohort characteristics

The study cohort was characterised according to self-reported health factors, including smoking status, counselling with their general practitioner due to flare-ups, and body mass index (BMI) classified according to the WHO as underweight, normal weight, overweight, or obese [23]. Medical records data were used to classify the cohort according to their previous hospital management, which included previous *surgery*, previous *inpatient* hospital contacts primarily due to diverticulosis, previous *outpatient* hospital contacts primarily due to diverticulosis, or *diverticulosis* as a secondary diagnosis only at any hospital contact. Furthermore, medical records data were used for the estimation of time from the first diagnosis of diverticulosis to questionnaire response, classifying disease severity (complicated if any contact involved an abscess/perforation, stenosis, or fistula and otherwise uncomplicated), and for the classification of comorbidity level according to the Charlson Comorbidity Index (CCI) [24].

### Patient-reported outcome measures

#### The DV-QOL questionnaire

The diverticulitis-specific DV-QOL questionnaire is a 17-item instrument covering four domains: physical symptoms (five items), concerns (three items), feelings (four items), and behavioural changes (five items) [4].

##### Scoring

The score of each DV-QOL domain is calculated by normalising the sum of the relevant items on a 0-to-10 scale, with lower scores indicating a better QOL. The DV-QOL total score is calculated by summarising the normalised scores of the four domains on a 0-to-10 scale, with each domain weighted equally, as outlined in the work by Khor et al. [25]. The patient’s acceptable symptom state (PASS) score has been established to 3.2, i.e. scores below 3.2 indicate an acceptable symptom state, while scores of 3.2 and above indicate a QOL-impacting symptom state [25].

#### The anchor QOL question

One anchor QOL question was added to the questionnaire for validation purposes: “Overall, how much does your bowel function affect your quality of life?” with the four mutually exclusive responses of “Not at all”, “A little”, and “Quite a bit” or “Very much”.

#### The EQ VAS measure

The EQ VAS score item is a rating of current generic health ranging from 0 to 100, with higher scores indicating better health [22]. The Danish population norm EQ VAS score has been previously determined to be 82 [26].

### Psychometric properties

#### Validity

The validity of a PROM is the degree to which the measurement assesses the construct it claims to measure [14]. It was evaluated in terms of two subtypes of *construct validity*: *convergent* and *discriminant* validity.

*Convergent validity* was assessed by calculating correlations between DV-QOL and the anchor QOL question and between the DV-QOL score and the EQ VAS score. To facilitate the analysis using the anchor QOL question, participants were categorised into three groups based on their responses: “Not at all” was classified as having no impact of bowel function on QOL, “A little” was classified as minor impact of bowel function on QOL, and “Quite a bit” or “Very much” were classified as some/major impact of bowel function on QOL.

*Discriminant validity* was assessed to investigate the ability of the DV-QOL score to differentiate between groups of subjects previously found to be likely to differ in terms of QOL. The groups explored were based on hospital management for diverticulosis, age, and sex.

#### Reliability

The reliability of a PROM is the degree to which it is free from measurement error [14]. The reliability of the DV-QOL score was assessed in terms of *internal consistency* by evaluating the inter-item correlation and the extent to which the items on each of the subscales of the DV-QOL score are interrelated [27].

#### Sensitivity and specificity

The sensitivity and specificity of the DV-QOL score in identifying responders with or without symptoms that impact QOL were investigated using the anchor QOL question.

### Statistical analysis

Descriptive statistics were compiled to characterise responders consenting to data linkage. Quantitative data were presented as medians with interquartile ranges (IQRs), and categorical data were presented as absolute numbers and percentages.

#### Missing data

Only responses with no missing data in the 17 DV-QOL items were included in the analysis.

#### Psychometric characteristics

The DV-QOL total score was computed, and three DV-QOL score groups were defined: those with a DV-QOL score below the PASS of < 3.2, indicating no QOL limiting disease; those with a DV-QOL score of 3.2–5, indicating minor QOL limiting disease; and those with a DV-QOL score > 5, indicating major QOL limiting disease.

*Convergent validity* in terms of the association between the DV-QOL total score and the anchor QOL question was evaluated as the correlation between the three groups previously defined for each of the two measures. The fit was considered perfect when responders reported no QOL-limiting disease on the DV-QOL total score and no impact of bowel function on QOL. Accordingly, minor QOL-limiting disease on the DV-QOL score and a minor impact of bowel function on QOL was a *perfect fit*, as well as major QOL-limiting disease on the DV-QOL score and some/major impact of bowel function on QOL. A difference by one category was considered a *moderate fit*, and *no fit* was considered if the difference was between two categories. The difference in the numerical DV-QOL score between the anchor QOL question groups was illustrated in a box plot and tested using the Kruskal–Wallis test.

Convergent validity was further explored by examining the relationship between the DV-QOL total score and the EQ VAS score. The association between the DV-QOL PASS score and the EQ VAS population norm score was explored and compared by applying Pearson’s Chi-squared test. Additionally, the median EQ VAS scores for the three total DV-QOL score groups were compared by using the Kruskal–Wallis test.

The *discriminative validity* was explored by comparing the groups defined by hospital management, age (younger or older than the median age of the study population, i.e. 70 years), and sex (male or female). The difference in the numerical DV-QOL scores between the groups was illustrated in a box plot and tested using the Wilcoxon rank sum test or the Kruskal–Wallis test.

*Reliability* was assessed by measuring average inter-item correlation and Cronbach’s α coefficient. The inter-item correlation measures the extent to which items on a scale assess the same construct, and a correlation between 0.2 and 0.5 is usually regarded as sufficient, while correlations > 0.7 indicate that items measure almost the same thing [28]. Cronbach’s α coefficient measures the interrelatedness of the items on a scale. The coefficient ranges from 0 to 1, with higher values indicating greater internal consistency, generally considered acceptable when between 0.7 and 0.9 [28].

The *sensitivity and specificity* of the DV-QOL score to identify responders with some/major impact of bowel function on QOL with a cutoff value of 3.2 (the PASS score) was explored in a receiver operating characteristic (ROC) curve analysis.

Statistical analyses were conducted using RStudio, version 2024.04.2 + 764 (Posit PBC) [29, 30].

## Results

### Translation and pilot testing

The 17 questions of the DV-QOL questionnaire were translated without experiencing significant problems. Discrepancies regarding the choice of words were cleared among the translators. The pilot test of the Danish version of the DV-QOL revealed that the questionnaire was straightforward for participants to understand. The Danish version of the DV-QOL is available in.

### Validation cohort characteristics

**Fig. 1:**
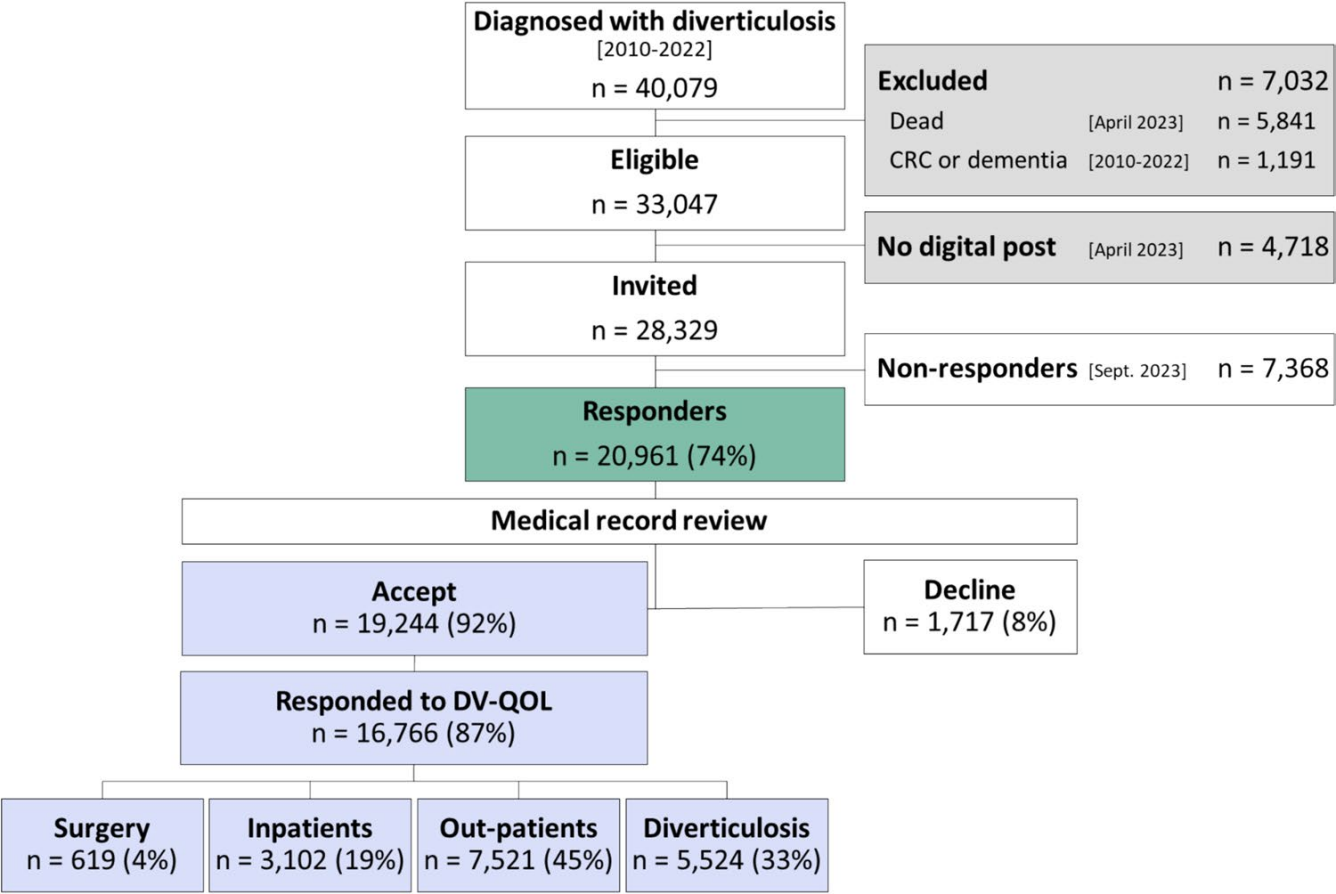
Study flow of patients

The target population comprised 40,079 subjects diagnosed with diverticulosis, of whom 28,329 subjects were invited to participate (Fig. 1). The target population had a median age of 74 (IQR 64–81) years, with 53% female, while eligible subjects had a median age of 71 (IQR 63–78) years, with 53% female. Of the 19,244 responders who accepted data linkage, 16,766 (87%) had complete answers to the entire DV-QOL instrument and, therefore, comprised the validation cohort. The 2478 responders who had not completed the entire DV-QOL had a median age of 75 years (IQR 68–80), and 51% were female. The median age of the validation cohort was 70.0 years (IQR 61.4–76.2), and 52% were women. Responders who had not completed the entire DV-QOL had the same frequency of complicated disease (4%) and the same time from diagnosis to survey (median 4, IQR 2–7 years), but were more likely to have severe comorbidity (19% vs. 12%) compared to responders who completed the entire DV-QOL.

The validation cohort was categorised into management groups as follows: 619 (4%) underwent *surgery*, 3102 (19%) were *inpatients*, 7521 (45%) were *outpatients*, and 5524 (33%) had *diverticulosis* (Table 1). Compared to outpatients and those with diverticulosis, responders who underwent surgery or with inpatient contacts were younger, more frequently female, and more likely to have complicated disease and to have sought counselling from their general practitioner due to flare-ups (Table 1).

**Table 1:** Validation cohort characteristics, median (IQR) or *n* (%)

		Management Group			
*N*	All	Surgery	Inpatient	Outpatient	Diverticulosis
	16,766	619 (3.7%)	3102 (18.5%)	7521 (44.9%)	5524 (32.9%)
Age	70.0 (62.4–76.2)	65.1 (56.4–73.1)	65.4 (57.3–73.3)	70.9 (63.8–77.0)	71.1 (64.7–76.5)
Sex					
Female	8642 (52%)	352 (57%)	1961 (63%)	3869 (51%)	2460 (45%)
Male	8124 (48%)	267 (43%)	1141 (37%)	3652 (49%)	3064 (55%)
CCI score					
0	9744 (58%)	375 (61%)	1918 (62%)	4323 (57%)	3128 (57%)
1–2	4933 (29%)	179 (29%)	846 (27%)	2262 (30%)	1646 (30%)
3 +	2089 (12%)	65 (11%)	338 (11%)	936 (12%)	750 (14%)
BMI (kg/m^2^)					
Underweight (< 18.5)	149 (0.9%)	5 (0.8%)	16 (0.5%)	80 (1.1%)	48 (0.9%)
Normal (18.5–24.9)	5314 (32%)	175 (29%)	882 (29%)	2568 (35%)	1689 (31%)
Overweight (25–29.9)	6706 (41%)	256 (42%)	1198 (39%)	2973 (40%)	2279 (42%)
Obese (30 +)	4302 (26%)	170 (28%)	965 (32%)	1768 (24%)	1399 (26%)
Smoking status					
Never	6699 (40%)	227 (37%)	1281 (41%)	3083 (41%)	2108 (38%)
Former	8205 (49%)	310 (50%)	1481 (48%)	3660 (49%)	2754 (50%)
Active smoker	1809 (11%)	81 (13%)	332 (11%)	758 (10%)	638 (12%)
Time from diagnosis to survey					
< 5 years	9064 (54%)	217 (35%)	1400 (45%)	3936 (52%)	3511 (64%)
≥ 5 years	7702 (46%)	402 (65%)	1702 (55%)	3585 (48%)	2013 (36%)
Disease severity					
Uncomplicated	16,042 (96%)	306 (49%)	2756 (89%)	7473 (99%)	5507 (100%)
Complicated	724 (4.3%)	313 (51%)	346 (11%)	48 (0.6%)	17 (0.3%)
Flare-up at general practitioner					
Never	13,334 (84%)	349 (60%)	1826 (61%)	6301 (88%)	4858 (94%)
Yes	2597 (16%)	231 (40%)	1173 (39%)	864 (12%)	329 (6.3%)

#### DV-QOL scores

The median DV-QOL total score was 2.5 (IQR 2.0–3.6). The median scores in the four subscales for the overall cohort ranged between 2.0 and 2.7, the highest on the symptoms scale (Table 2). Overall, 11,466 (68%) reported a DV-QOL total score under the PASS score, indicating an acceptable symptom state, with significant differences between the groups (Table 2).

**Table 2:** DV-QOL score for the overall cohort and the management groups, n (%) or median (IQR)

			Management Group			
N	All		Surgery	Inpatient	Outpatient	Diverticulosis
		16,766	619 (3.7%)	3,102 (18.5%)	7,521 (44.9%)	5,524 (32.9%)
Domain	Symptoms	2.7 (1.7–4.0)	3.3 (2.0–5.0)	3.3 (2.0–4.3)	2.7 (1.7–4.0)	2.3 (1.7–3.7)
	Concerns	2.0 (2.0–4.0)	3.3 (2.0–4.7)	2.7 (2.0–4.0)	2.0 (2.0–4.0)	2.0 (2.0–3.3)
	Feelings	2.0 (2.0–3.5)	3.5 (2.0–5.0)	2.8 (2.0–4.0)	2.0 (2.0–3.5)	2.0 (2.0–3.0)
	Behaviour	2.0 (2.0–3.2)	3.2 (2.0–4.8)	2.4 (2.0–3.6)	2.0 (2.0–3.2)	2.0 (2.0–2.8)
DV-QOL total score		2.5 (2.0–3.6)	3.4 (2.3–4.8)	2.9 (2.2–4.0)	2.5 (2.0–3.5)	2.3 (1.9–3.1)
Patient acceptable symptom state (DV-QOL total score < 3.2)						
Acceptable		11,466 (68%)	290 (47%)	1,811 (58%)	5,147 (68%)	4,218 (76%)
Unacceptable		5,300 (32%)	329 (53%)	1,291 (42%)	2,374 (32%)	1,306 (24%)

### Psychometric properties

#### Convergent validity

**Fig. 2:**
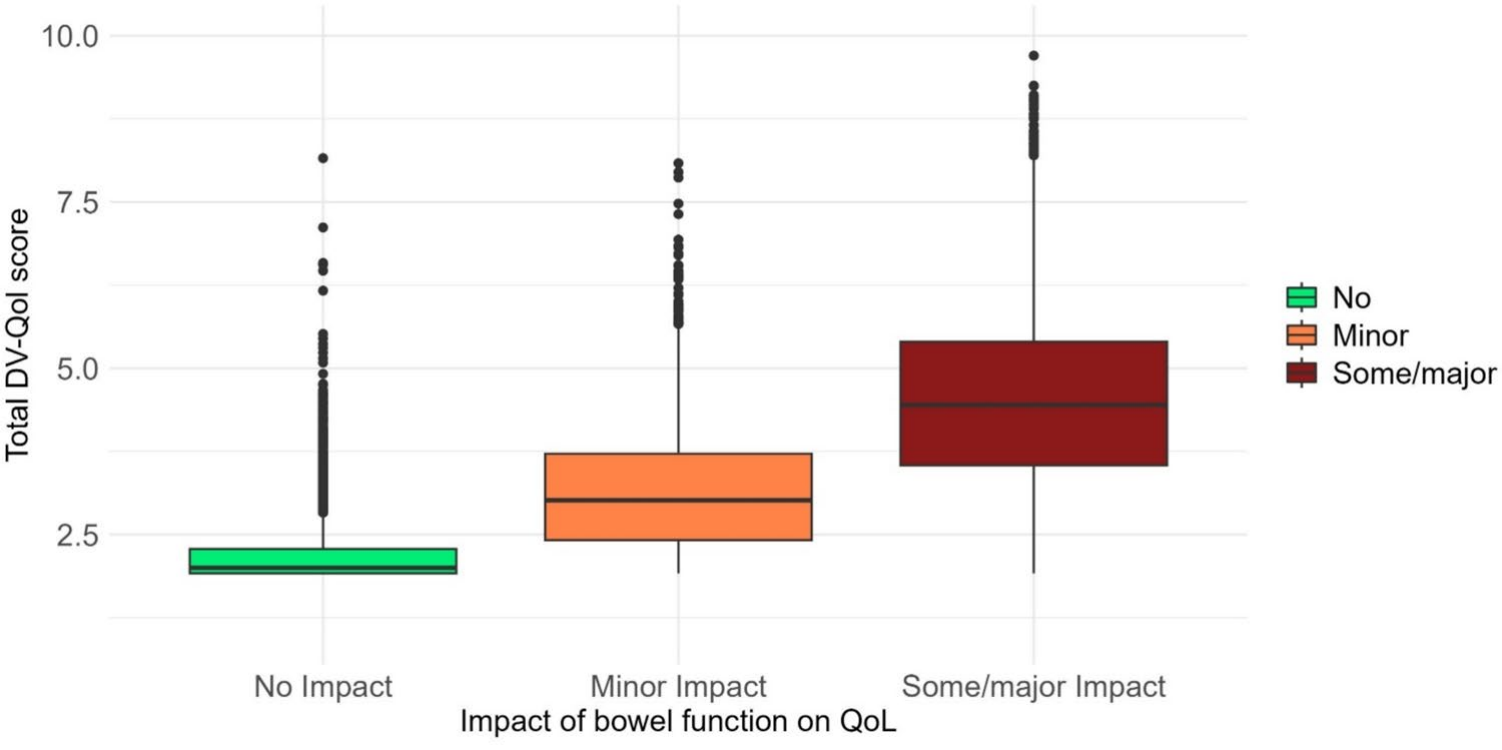
Boxplot illustrating the association between the median DV-QOL total score and the impact of bowel function on quality of life (QOL)

The proportion of responders with a perfect fit between the DV-QOL category and the anchor QOL question was 66%; a moderate fit was found in 32%, and no fit in 3% (Table 3). For responders reporting no impact of bowel function on QOL (*n* = 7,731), the median DV-QOL total score was 2 (IQR 1.9–2.3), for those reporting a minor impact of bowel function on QOL (*n* = 5828), the median DV-QOL total score was 3.0 (IQR 2.4–3.7), and for those reporting some/a major impact on QOL (*n* = 2985), the median DV-QOL total score was 4.5 (IQR 3.5–5.4) (Fig. 2). Differences in the DV-QOL total score between QOL categories were statistically significant (*P* < 0.001).

**Table 3:** Fit between the DV-QOL total score category and the anchor QOL question, *n* (%)

		Impact of bowel function on QOL			
		No		Minor	Some/major
*N* (%)		7731 (46%)		5828 (35%)	2985 (18%)
DV-QOL total score (QOL limiting diverticular disease)	< 3.2 (No)	11,466 (68%)	7427 (45%)	3321 (20%)	542 (3%)
	3.2–5 (Minor)	4168 (25%)	292 (2%)	2362 (14%)	1478 (9%)
	> 5 (Major)	1132 (7%)	12 (0%)	145 (1%)	965 (6%)

Of the 5300 responders with QOL limiting diverticular disease (i.e. DV-QOL total score ≥ 3.2), 3774 (74%) accordingly reported an EQ VAS score below the population norm of 82. In comparison, 6069 (55%) of the 11,466 responders with a DV-QOL total score < 3.2 reported an EQ VAS score at or above the population norm score (*p* < 0.001).

**Fig. 3:**
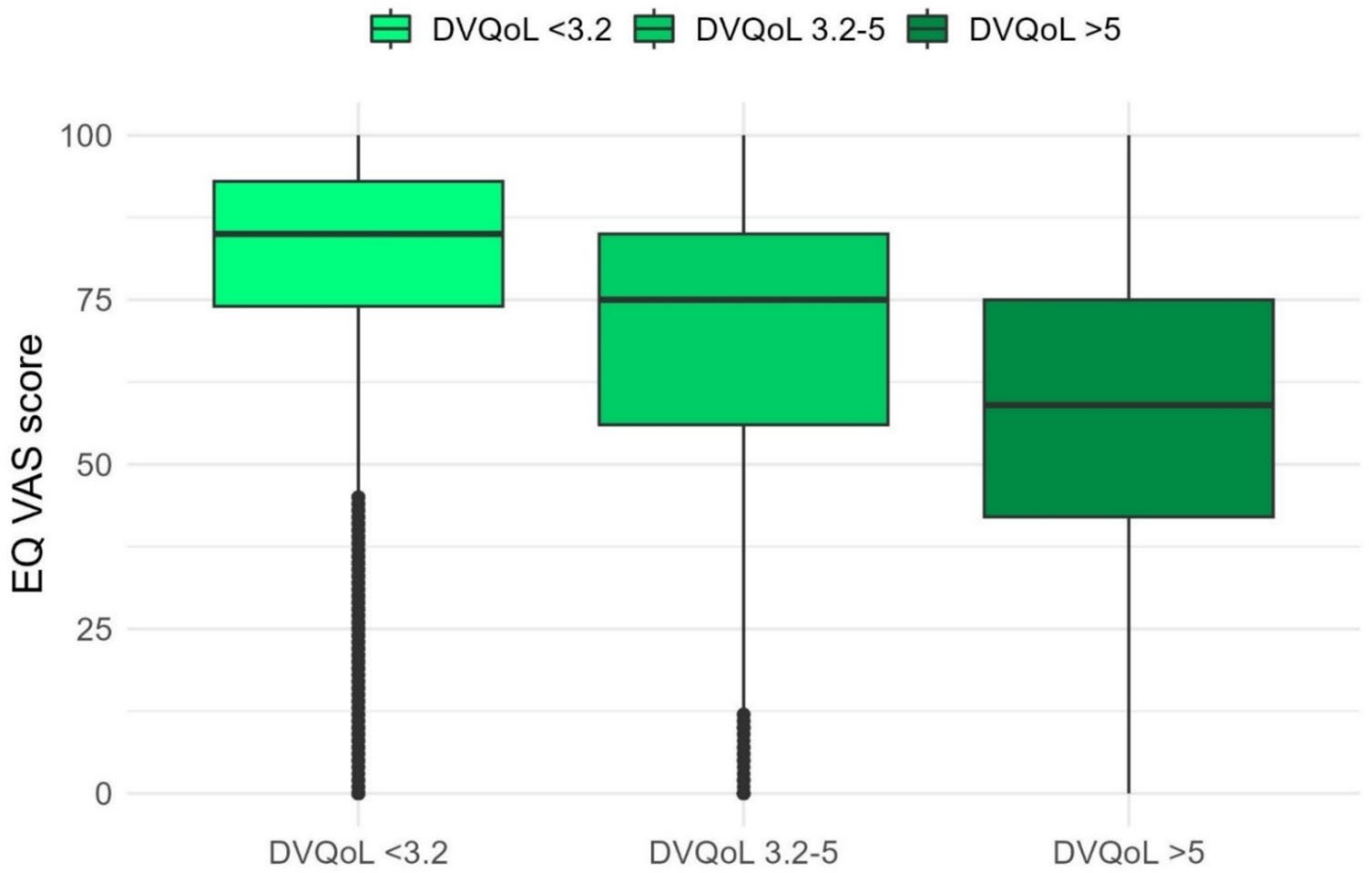
Boxplot showing median (bold line), interquartile range (coloured area), and range of the EQ VAS score according to DV-QOL score category

Responders reporting no QOL limiting diverticular disease (*n* = 11,466) accordingly had the highest EQ VAS score with a median of 85 (IQR 74–93); those with minor DV-QOL impact (*n* = 4,168) had EQ VAS score of 75 (IQR 56–85), and those reporting major QOL-limiting diverticular disease (*n* = 1132) accordingly reported the lowest EQ VAS score with a median of 59 (IQR 42–75) (Fig. 3). Differences in the EQ VAS score between DV-QOL score categories were statistically significant (*p* < 0.001).

#### Discriminative validity

**Fig. 4:**
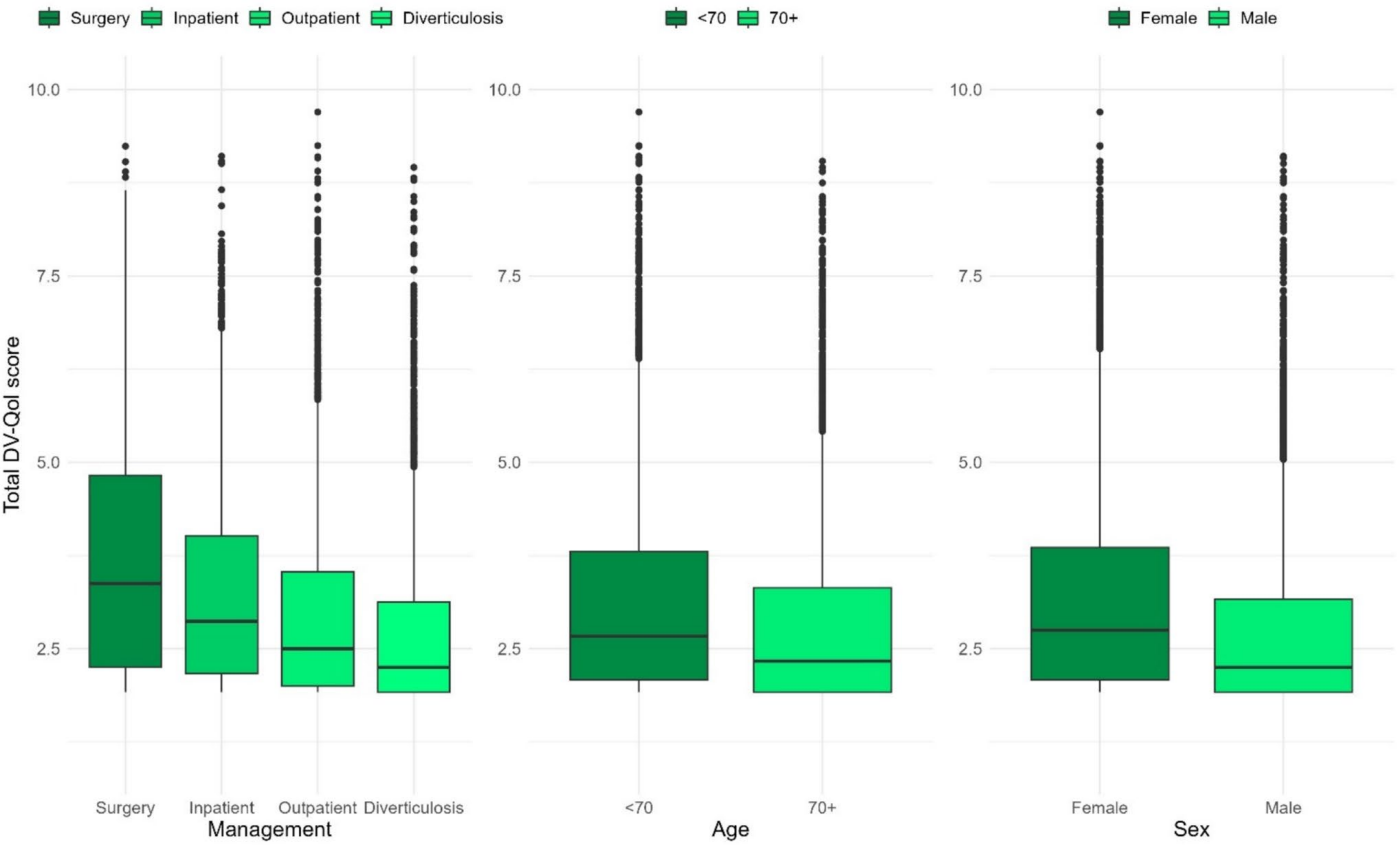
Comparison of median DV-QOL total score in groups of responders differing regarding disease management, age below or above the median, and sex

DV-QOL total median scores according to management groups were 3.4 (IQR 2.3–4.8) for those with *surgery*, 2.9 (IQR 2.2–3.5) for *inpatients*, 2.5 (IQR 2.0–3.5) for *outpatients*, and 2.3 (IQR 1.9–3.1) for those with *diverticulosis*, respectively. DV-QOL total median scores were 2.3 (IQR 1.9–3.3) for those aged ≥ 70 years and 2.7 (IQR 2.1–3.8) for those aged < 70 years, while females had a median score of 2.8 (IQR 2.1–3.9) and males 2.3 (IQR 1.9–3.2) (Fig. 4). The differences in DV-QOL total median scores were statistically significant between management groups (*p* < 0.001), age groups (*p* < 0.001), and sex (*p* < 0.001).

#### Reliability

The DV-QOL total score inter-item correlation was 0.41, ranging from 0.39 to 0.77 across the four domains. The Cronbach’s α coefficient was 0.92 for the DV-QOL total score and ranged between 0.76 and 0.91 for the four domains (Table 4).

**Table 4:** Inter-item correlation and Cronbach’s α coefficient

DV-QOL domain	Mean score	Number of items	Cronbach’s α	Average inter-item correlation
Physical symptoms	3.1	5	0.76	0.39
Concerns	2.9	3	0.91	0.77
Feelings	2.9	4	0.81	0.52
Behavioural changes	2.8	5	0.82	0.49
DV-QOL total score	2.9	17	0.92	0.41

#### Sensitivity and specificity

**Fig. 5:**
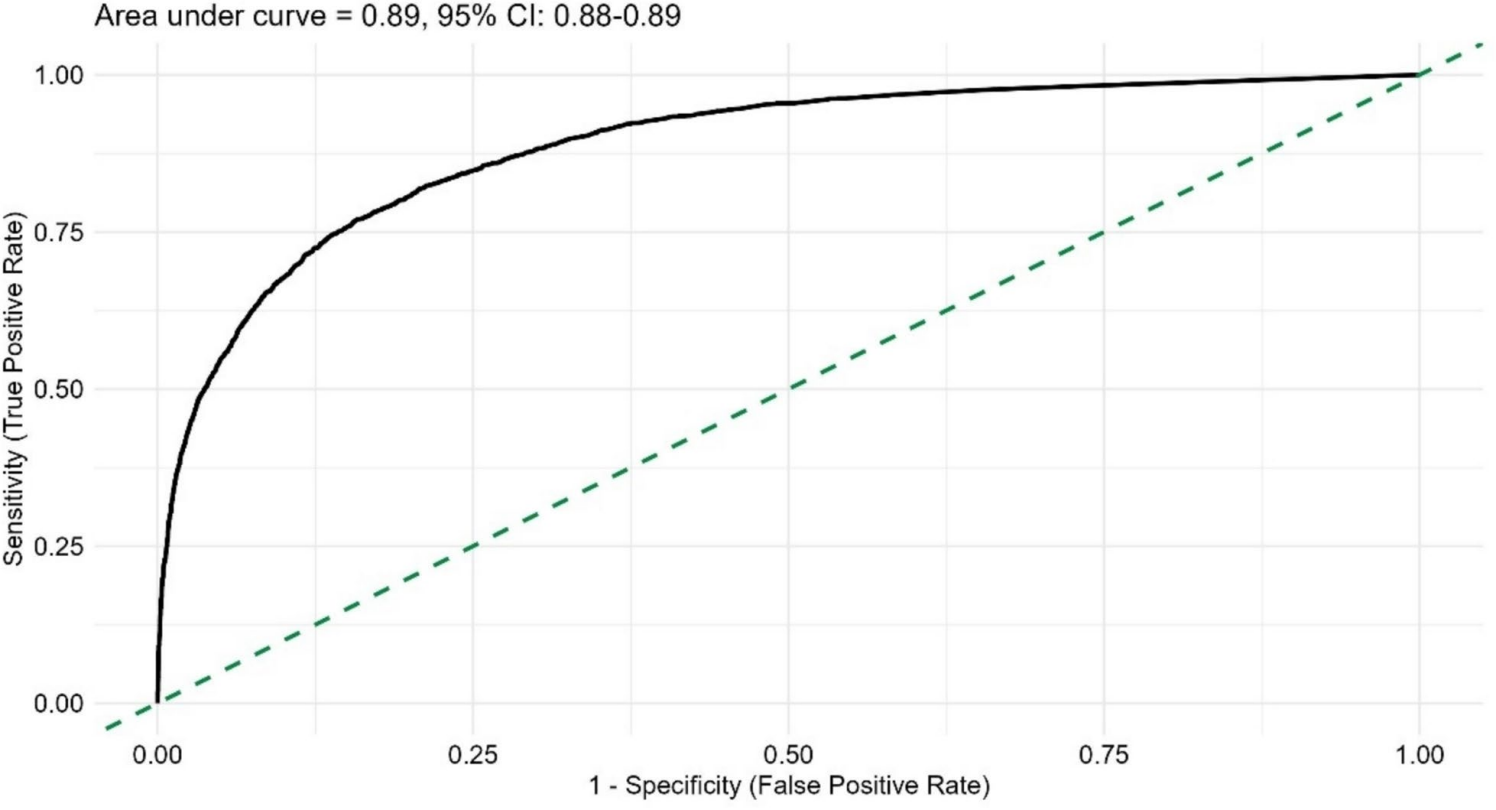
Receiver operating characteristic (ROC) curve

ROC curve analyses showed that with a cutoff of 3.2 points, the DV-QOL score had a sensitivity of 82% and a specificity of 79% for identifying responders with a *major* impact of bowel function on QOL. The area under the curve was 0.89 (95% CI 0.88–0.89) (Fig. 5).

If the cutoff of 3.2 points was used to identify patients with *minor and major* impact of bowel function on QOL, the sensitivity decreased to 56% while the specificity increased to 96%.

## Discussion

The Danish translation of the DV-QOL score was found to possess good psychometric properties in a large cohort of Danish subjects diagnosed with diverticular disease. The Danish version demonstrated high convergent and discriminant validity, as well as good reliability, with all items behaving consistently. The sensitivity and specificity were approximately 80% for identifying subjects with a major impact on bowel function on quality of life (QoL). Thus, our study supports the use of DV-QOL in evaluating diverticular disease-specific quality of life in Danish subjects with diverticulosis.

This study extends previous validations of the DV-QOL by including individuals with diverticulosis as a secondary diagnosis, presumed to be asymptomatic. Accordingly, the median scores across all four DV-QOL domains and the total scores were lower than in previous studies, likely due to the inclusion of individuals with less severe disease. The DV-QOL was developed for patients with symptomatic uncomplicated diverticular disease following acute diverticulitis and a similar cohort was used to establish the PASS score [4, 25]. It was demonstrated that subjects with diverticulosis, even without clinically overt symptoms, experience a wide range of health consequences [4]. Our findings indicate that the DV-QOL can be reliably applied to individuals without a history of acute diverticulitis. Previous validations have been conducted in German-speaking patients from Switzerland [31] and Italian-speaking patients from Italy [32], including patients with recurrent acute uncomplicated diverticulitis or elective surgery for recurrent disease. Our findings align with prior studies, supporting the applicability of the DV-QOL.

The Danish version of the DV-QOL demonstrated high internal consistency, with a Cronbach’s α of 0.92, exceeding that of the German version (0.89) [31] and slightly lower than the original English version (0.95) [4]. The total score and three of the four domains showed inter-item correlation coefficients ranging from 0.39 to 0.52, indicating sufficient unique variance. The concerns domain had a higher inter-item correlation (0.77), indicating substantial overlap among items, which is consistent with findings from the German validation (0.74) [31].

While the anchor QOL question specifically assessed the impact of bowel function on QOL, the EQ VAS score provided a measure of generic QOL. The Danish DV-QOL exhibited expected correlations with the anchor QOL question and the EQ VAS score. Among responders, 32% showed only a moderate fit between the DV-QOL total score and the anchor QOL question, primarily due to reporting a minor impact of bowel function on QOL despite having a DV-QOL total score < 3.2. ROC curve analyses indicated that the 3.2-point cutoff had 82% sensitivity for identifying individuals with a *major* impact of bowel function on QOL but only 56% sensitivity for detecting those with both *minor and major* impacts, suggesting that the threshold may be too high to capture patients with mild symptoms. However, the increased specificity of 96% indicated that the cutoff effectively excludes individuals without impact. The observed correlation between DV-QOL and EQ VAS supports the convergent validity of the DV-QOL, confirming its ability to capture broader health-related QOL, including psychological, mental, and social aspects.

The availability of the Danish DV-QOL provides clinicians and researchers with a PROM for assessing health-related QOL in Danish subjects with diverticulosis. This facilitates the evaluation of disease burden and patient outcomes, which is particularly important for a benign yet potentially disabling condition, where management guidelines emphasise an individualised, patient-centred approach that integrates QOL with other clinical measures [9, 10, 33]. The DV-QOL captures the emotional impact of diverticulosis, with questions designed to ensure respondents attribute their symptoms specifically to the condition. This enhances the tool’s relevance in assessing disease burden. Many challenges in managing diverticulosis arise from a limited understanding of its functional and psychosocial impact. Addressing this gap is essential for developing comprehensive management strategies, and a valid, reliable, disease-specific QOL instrument is a crucial first step.

Our validation cohort excluded subjects without digital mail, which may have resulted in an underrepresentation of older subjects and those with lower socioeconomic status, potentially introducing selection bias and limiting generalisability. However, the cohort had a higher median age (70 years) than previous validation studies (57–62 years) [4, 25, 31] and included a greater proportion of individuals younger than 57 years due to its larger sample size. Although electronic distribution may have precluded some individuals with lower socioeconomic status, 88% of Danish residents use digital mail [18], mitigating this limitation. Nonetheless, incorporating data on educational and socioeconomic status would have strengthened the assessment of the tool’s generalisability.

### Strengths and limitations

A key strength of this study is the large cohort size and high response rate, which enhance the generalisability of the findings and reduce the risk of selection bias. The validation cohort encompassed a broad spectrum of subjects, ranging from those presumably asymptomatic to those with chronic symptoms and from those managed outpatient to those who had undergone surgery, as well as individuals across a wide age range and with varying disease durations. This diversity strengthens the applicability of the Danish DV-QOL across different patient profiles.

However, certain limitations should be acknowledged. Convergent validity was assessed using an anchor QOL question that has not been externally validated, which may introduce some uncertainty regarding its comparability to established health-related QOL measures. Additionally, test–retest reliability could not be evaluated due to the absence of a follow-up assessment, limiting the ability to determine the stability of responses over time. Furthermore, while digital distribution enabled efficient data collection, it may have excluded individuals without access to digital mail, potentially affecting the representativeness of the sample, particularly among older adults and those with lower socioeconomic status.

## Conclusions

This study validated the Danish version of the DV-QOL, demonstrating strong psychometric properties, including high internal consistency, construct validity, and reliability. The tool effectively captured the health-related QOL in subjects with diverticulosis, including those without a history of acute diverticulitis. The availability of a validated Danish DV-QOL provides clinicians and researchers with a reliable instrument for assessing disease burden and patient outcomes, contributing to a more comprehensive and patient-centred approach to managing diverticulosis. Future research should focus on further evaluating the responsiveness of the DV-QOL over time.

## Supplementary Information

Below is the link to the electronic supplementary material.

Supplementary file1 (PDF 181 KB)

## Data Availability

No datasets were generated or analysed during the current study.
