# Supplementary material for: Quality of life in diverticular disease: translation and validation of the Danish version of the diverticulitis quality of life instrument (DV‑QOL)
Source: Int J Colorectal Dis. 2025 May 14;40(1):117. doi: 10.1007/s00384-025-04911-z (PMC12078394; doi:10.1007/s00384-025-04911-z)
Supplement: Supplementary file 1 — Supplementary file1 (PDF 181 KB) [file 384_2025_4911_MOESM1_ESM.pdf]

SPØRGESKEMA TIL

LIVSKVALITET MED DIVERTIKLER (UDPOSNINGER PÅ TYKTARMEN) (DV-QOL)

Vejledning

Dette spørgeskema spørger dig om din livskvalitet, eller "hvordan du har det" med divertikulose (udposninger på tyktarmen). Der er spørgsmål til, hvordan divertikler kan påvirke dig fysisk, følelsesmæssigt og socialt. Besvar alle spørgsmålene så godt du kan. Hvis du er usikker på et svar, så skriv et kvalificeret gæt. Der findes ingen rigtige eller forkerte svar på disse spørgsmål. Når du besvarer spørgsmålene, skal du kun tænke på dine oplevelser med divertikler i løbet af de **sidste 2 uger.**

Mange tak!

Nogle mennesker rapporterer, at de har episoder med mavesmerter *forårsaget af deres divertikler* – dvs. smerter inden for det røde område vist på billedet nedenfor. Disse episoder opstår når mavesmerter kommer pludseligt eller bliver værre end sædvanligt. Nedenfor findes spørgsmål om disse episoder. Når du besvarer disse spørgsmål, skal du kun tænke på de sidste 2 uger.

For disse spørgsmål betyder "mave" det røde område nedenfor.

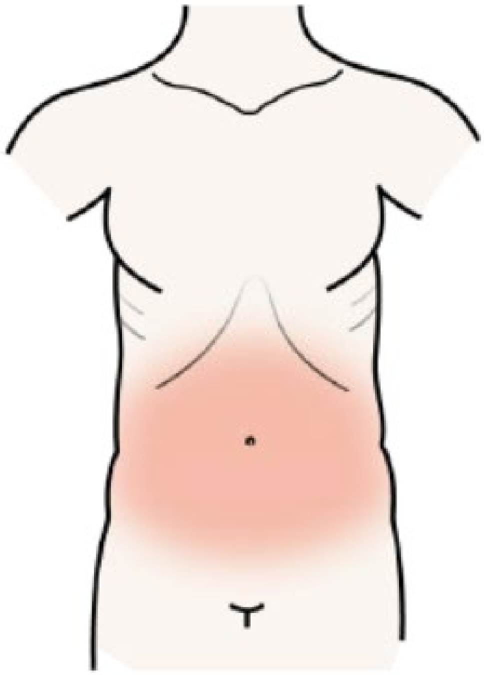

Disse spørgsmål spørger til almindelige mavesymptomer, i løbet af de sidste 2 uger. Tænk over alle de mavesymptomer, du har haft, *der er forårsaget af dine divertikler*, når du besvarer disse spørgsmål.

Nedenfor findes en liste med symptomer Læs dem alle og marker det felt, der bedst beskriver hvor mange dage du havde det sådan i løbet af de sidste 2 uger. Marker kun ét felt for hvert spørgsmål og spring ikke nogen spørgsmål over. Det er ok at komme med et kvalificeret gæt, hvis du er usikker på nøjagtige antal dage du havde symptomet. Hvis du ikke havde det sådan i de sidste 2 uger, markeres "ingen dage".

	I de sidste 2 uger, hvor mange dage ...	Ingen dage	Kun 1 dag	2-5 dage	10-13 dage	Hver dag
S1	Følte du dig oppustet – dvs. følte du at maven var fuld?					
S3	Havde du tynd eller vandig afføring?					
S6	Følte du trang til at have afføring, men kunne ikke komme af med det hele?					
S7	Havde du kvalme – dvs. følte at du skulle kaste op?					
S8	Havde du mavesmerter?					

➔ Hvis du svarede "kun 1 dag" eller mere for mavesmerter (spørgsmål S8), så skal du besvare spørgsmål S9 nedenfor. Ellers kan du springe til det næste afsnit.

S9. Nedenfor findes et billede, der viser forsiden af maven. Maven er opdelt i 9 områder, nummereret fra "1" til "9". Vælg området hvor du følte dine divertikel-mavesmerter, mindst én gang i løbet af de sidste 2 uger. Du kan vælge mere end et område, hvis du havde smerter i mere end et område.

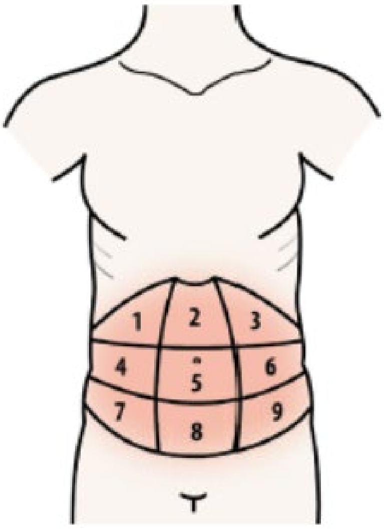

Område 1 [ ]   Område 2 [ ]   Område 3 [ ]
Område 4 [ ]   Område 5 [ ]   Område 6 [ ]
Område 7 [ ]   Område 8 [ ]   Område 9 [ ]

Nedenfor findes en liste over mulige bekymringer vedr. divertikler. Læs hver udtalelse og marker det felt der bedst beskriver, hvor meget du følte den bekymring, hvis relevant, i løbet af de sidste 2 uger. Marker kun ét felt for hver udtalelse og spring ikke nogen spørgsmål over. Hvis du overhovedet ikke har haft det sådan i løbet af de sidste 2 uger, markeres "Slet ikke bekymret".

	I de sidste 2 uger, hvor bekymret var du for at...	Slet ikke bekymret	Lidt bekymret	Rimeligt bekymret	Ret bekymret	Meget bekymret
C1	Dine divertikler kunne blusse op eller blive værre når som helst					
C3	Der kunne være noget alvorligt galt med din krop, på grund af dine divertikler					
C6	Dine divertikler forårsagede skade i din krop					

Nedenfor findes en liste over udtalelser om, hvordan divertikler kan få dig til at føle dig socialt eller følelsesmæssigt. Læs dem alle og marker det felt, der bedst beskriver hvor ofte du havde det sådan i de sidste 2 uger. Marker kun ét felt for hver udtalelse og spring ikke nogen spørgsmål over. Hvis du overhovedet ikke havde det sådan i de sidste 2 uger, markeres "På intet tidspunkt".

	I de sidste 2 uger ...	På intet tidspunkt	Sjældent	Nogle gange	Ofte	Hele tiden
F2	Jeg følte at andre så ned på mig, på grund af symptomer fra mine divertikler					
F4	Jeg følte mig frustreret på grund af mine divertikler					
F6	Jeg følte mig urolig på grund af mine divertikler					
F7	Jeg følte mig irriteret på grund af mine divertikler					

Nedenfor findes en liste over udtalelser om, hvordan divertikler kan påvirke, hvad du foretager dig. Læs dem alle grundigt og marker det felt, der bedst beskriver hvor ofte, hvis nogensinde, du opførte dig sådan i de sidste 2 uger. Marker kun ét felt for hver udtalelse og spring ikke nogen spørgsmål over. Hvis du overhovedet ikke havde det sådan i de sidste 2 uger, markeres "På intet tidspunkt".

	I de sidste 2 uger ...	På intet tidspunkt	Sjældent	Nogle gange	Ofte	Hele tiden
B2	Jeg spiste mindre mad end sædvanligt på grund af mine divertikler					
B4	Jeg var nødt til at gå med løstsiddende tøj på grund af mine divertikler					
B6	Jeg var nødt til at undgå sociale begivenheder på grund af mine divertikler					
B10	Jeg kunne ikke sove på grund af mine symptomer fra mine divertikler					
B11	Jeg måtte gå glip af arbejde eller andre vigtige forpligtelser på grund af mine divertikler					
